# Lysine lactylation in glioma: from metabolic stress to epigenetic remodeling and therapeutic resistance

**DOI:** 10.3389/fcell.2026.1821274

**Published:** 2026-10-12

**Authors:** Xiaosong Hu, Li Yang, Yanghao Hu, Hongjuan Cui

**Affiliations:** 1 State Key Laboratory of Resource Insects, Medical Research Institute, Southwest University, Chongqing, China; 2 Jinfeng Laboratory, Chongqing, China

**Keywords:** epigenetic remodeling, glioma, lactate, lysine lactylation (Kla), metabolic reprogramming, therapeutic resistance

## Abstract

Glioma is characterized by profound metabolic reprogramming and sustained lactate accumulation; however, the mechanisms by which lactate-rich metabolic stress is translated into stable malignant phenotypes remain incompletely understood. Lysine lactylation (Kla), a recently identified post-translational modification, has emerged as a critical nexus linking lactate metabolism to durable epigenetic and protein-state adaptations. In glioma, accumulating evidence indicates that Kla contributes to multiple dimensions of tumor biology, including oncogenic transcriptional activation, glioma stem-like cell (GSC) maintenance, immunosuppressive remodeling, and therapeutic resistance. Mechanistically, histone Kla rewires metabolic stress-responsive transcriptional programs, whereas non-histone Kla executes rapid, transcription-independent responses by directly modifying key regulatory proteins involved in DNA repair, signal transduction, and metabolic adaptation. This review systematically summarizes current progress on the lactate-Kla axis in glioma, encompassing the basis of lactate metabolism, the molecular regulatory network of Kla, and the distinct yet coordinated functions of histone and non-histone lactylation. We further discuss how Kla contributes to temozolomide (TMZ) resistance, radioresistance, and chemoradiotherapy adaptation, and outline a hierarchical therapeutic framework targeting this pathway. Despite rapid advances, important areas requiring further investigation include subtype-specific variation in lactate–Kla biology and the potential crosstalk between Kla and other post-translational modifications. A better understanding of these issues will help define the biological significance of the lactate–Kla axis and guide the future development of lactylation-based therapeutic strategies in glioma.

## 1 Introduction

Glioma is the most common primary malignant neoplasm of the central nervous system, and glioblastoma (GBM) represents its most aggressive adult form (Stupp et al., 2009; Wang C. et al., 2022; Vuong et al., 2018; Schaff and Mellinghoff, 2023). Despite maximal safe resection followed by radiotherapy and chemotherapy with temozolomide (TMZ), most patients experience recurrence, underscoring the remarkable capacity of glioma cells to adapt under sustained therapeutic stress (Tang et al., 2019; Yan et al., 2024; Mishchenko et al., 2025). One of the hallmarks of this adaptation is metabolic reprogramming, particularly aerobic glycolysis and excessive lactate accumulation (Zhao et al., 2023; Zeng et al., 2022; Lu et al., 2022). Lactate, traditionally known as a metabolic waste product, has recently emerged as a versatile metabolic messenger that can influence chromatin regulation, intercellular communication, and tumor-immune interactions (Rabinowitz and Enerback, 2020; Zhang et al., 2019; Dai et al., 2023). However, how lactate accumulation is translated into stable molecular programs that reinforce malignant progression and treatment tolerance remains poorly understood.

The discovery of lysine lactylation (Kla) provides a vital mechanistic bridge between lactate abundance and durable epigenetic or protein-state adaptation (Hagihara et al., 2021; Wang J. et al., 2024). Kla may be viewed as a molecular “decoder” of lactate-induced stress and, more precisely, as a metabolic–epigenetic coupling mechanism. At the chromatin level, histone Kla links metabolic flux to transcriptional remodeling, thereby establishing transcriptional states permissive for stemness maintenance and immune evasion (Jian et al., 2026; Liu Y. et al., 2025; Ma et al., 2025). Conversely, non-histone Kla acts as a rapid, transcription-independent sensor that directly modulates protein stability, enzymatic activity, and signaling cascades, executing immediate DNA repair and metabolic rewiring under chemoradiotherapy stress (Jin et al., 2023; Zhang et al., 2025; Peng and Du, 2025). In glioma, these effects are particularly relevant to glioma stem-like cell (GSC) maintenance and treatment adaptation (Wang S. et al., 2024). In this sense, lactylation serves as a metabolic-epigenetic coupling factor that converts transient metabolic perturbations into persistent adaptive tumor programs.

This Review summarizes the current understanding of lactate metabolism and the molecular basis of Kla in glioma, then discusses the biological functions of histone and non-histone lactylation, their contributions to therapeutic resistance, and the translational potential of targeting the lactate-Kla axis. We further highlight the key unresolved questions that must be addressed before this emerging framework can be translated into clinically actionable strategies.

## 2 Lactate metabolism and the biochemical basis of protein lactylation in glioma

### 2.1 Lactate production, transport, and accumulation in glioma

Glioma cells exhibit significant metabolic reprogramming (Strickland and Stoll, 2017; Fan et al., 2026). Even under aerobic conditions, they maintain high glycolytic flux and sustained lactate production (Maher et al., 2012; Marin-Valencia et al., 2012). This feature supports rapid ATP generation and the provision of anabolic intermediates required for proliferation, while also enhancing the conversion of pyruvate to lactate, resulting in high intracellular lactate levels and prominent extracellular lactate accumulation (Vander Heiden et al., 2009; Lunt and Vander Heiden, 2011; DeBerardinis and Chandel, 2016). The lactate concentration gradient is further exacerbated by aberrant vasculature and diffusion barriers in hypoxic and perinecrotic tumor regions (Wu et al., 2026). A variety of oncogenic and microenvironmental signals together contribute to the lactate-rich microenvironment of glioma: hypoxia-induced signaling, especially HIF-1α, and oncogenic transcriptional programs upregulate glycolytic enzymes and lactate-generating pathways, thereby increasing lactate output under oxygen limitation and therapeutic stress (Wolf et al., 2011; Gabriely et al., 2017). In parallel, glioma cells rely on lactate-handling systems to maintain redox balance and mitigate acid stress, while also using lactate as a metabolic and signaling intermediate to coordinate adaptive cellular responses (Brooks, 2018; Faubert et al., 2017; Levy et al., 2024).

Lactate transport is a central component of glioma adaptation. Monocarboxylate transporters (MCTs), especially MCT1 and MCT4, mediate transmembrane lactate transport and enable its shuttling between tumor subpopulations as well as between tumor cells and the tumor microenvironment (TME) (Chen et al., 2025; Payen et al., 2020). Lactate flux therefore functions not only as a mode of metabolite exchange, but also as an intercellular communication channel that reshapes immune surveillance and reinforces malignant cell states (Brooks, 2018). For example, lactate derived from tumor-associated macrophages (TAMs) can be exported through MCT4 and imported into GSCs through MCT1, forming a TAM-tumor metabolic axis that shapes innate immune sensing and promotes the establishment of an immunosuppressive niche (Li et al., 2026). In addition, lactate can participate in transcriptional and phenotypic adaptation through hydroxycarboxylic acid receptor 1 (HCAR1)-dependent signaling, confirming that lactate is a bioactive metabolite rather than a simple metabolic waste product (He et al., 2025; Longhitano et al., 2022). Lactate accumulation in glioma is also not solely determined by glucose metabolism, because alternative carbon sources such as glutamine-supported anaplerosis influence glycolytic dependence and lactate dynamics, while therapy-induced stress further alters carbon utilization and lactate handling (Yang et al., 2009; McBrayer et al., 2018; Portais et al., 1996). Collectively, lactate levels in glioma represent an integrated output of oncogenic signaling, hypoxia, nutrient availability, and therapeutic stress, thereby providing a biochemical basis for downstream lactate-responsive regulatory processes such as lysine lactylation.

### 2.2 Biochemical basis and regulatory machinery of protein lactylation

Protein lactylation is an acylation modification in which a lactyl group is covalently attached to the ε-amino group of lysine residues (Zhang et al., 2019; Ren et al., 2025; Iozzo et al., 2025). At present, Kla has been detected in histone tails as well as in a wide range of non-histone proteins, suggesting broad regulatory potential in chromatin regulation, protein stability, and signal transduction (Duan et al., 2024; Wang et al., 2023; Meng et al., 2024). In glioma, the lactate-rich microenvironment generated by glycolytic reprogramming, hypoxia, and therapeutic stress further confers particular biological relevance on Kla (Wang S. et al., 2024). Since the initial report of histone lysine lactylation in 2019, a regulatory framework analogous to other acyl marks has been proposed, consisting of enzymatic “writers,” “erasers,” and “readers” (Song et al., 2026; Sheng et al., 2026). In the discovery study, p300/CBP was suggested as a candidate writer for histone Kla based on cellular perturbation experiments and *in vitro* activity assays (Zhang et al., 2019). Subsequent work expanded the writer landscape beyond p300/CBP. For example, GCN5 has been implicated in promoting histone lactylation-linked reparative transcriptional programs in monocytes/macrophages (Wang N. et al., 2022). In cancer-relevant contexts, KAT8 was reported to act as a pan-Kla writer that installs lactyl marks on multiple non-histone substrates, including eEF1A2, thereby enhancing translation and colorectal carcinogenesis (Xie et al., 2024). HBO1 was identified as a lysine lactyltransferase that catalyzes histone H3K9la and drives Kla-dependent transcription (Niu et al., 2024). In addition, aminoacyl-tRNA synthetases have emerged as lactyltransferases: AARS1 functions as a lactate sensor and lactyltransferase that catalyzes widespread protein lactylation, including p53, in tumor cells, whereas AARS2 has been reported as a mitochondrial protein lysine lactyltransferase that restrains OXPHOS under hypoxia (Zong et al., 2024; Mao et al., 2024).

Kla is also reversible. A systematic enzymology screen identified class I HDAC1-3 and SIRT1-3 as efficient delactylases *in vitro*, with HDAC1/3 showing site-specific delactylase activity in cells (Moreno-Yruela et al., 2022). Beyond these core delactylases, certain contexts have revealed enzyme-specific delactylation relationships; for instance, HDAC8 has been proposed as a delactylase for PRMT1 in hypoxia-driven metastasis models (Zhou J. et al., 2025). In parallel, emerging studies indicate that some deacetylases may also catalyze lactylation rather than remove it; for example, HDAC6 was reported to catalyze α-tubulin lactylation in neurons, thereby linking lactate availability to microtubule dynamics (Sun et al., 2024). Reader proteins remain the least defined component of the Kla regulatory machinery, particularly in glioma. Notably, currently reported Kla readers have been identified primarily in biological systems outside glioma. For example, Brg1 was reported to recognize H3K18la during early induced pluripotent stem cell reprogramming (Hu et al., 2024). High-throughput lactylome profiling further underscores that Kla extends far beyond histones. In hepatocellular carcinoma, an integrative lactylome/proteome study identified 9,275 Kla sites, with the vast majority mapped to non-histone proteins (Yang et al., 2023). In gastric cancer cells, proteomics identified 2,375 Kla sites across 1,014 proteins and suggested that elevated Kla abundance in tumors correlates with poorer prognosis (Yang et al., 2022). Together, these datasets support the view that protein lactylation is a widespread modification with potential functional roles in transcription, RNA processing, proliferation, and stress adaptation-processes central to tumor progression and therapy resistance (Figure 1).

**FIGURE 1:**
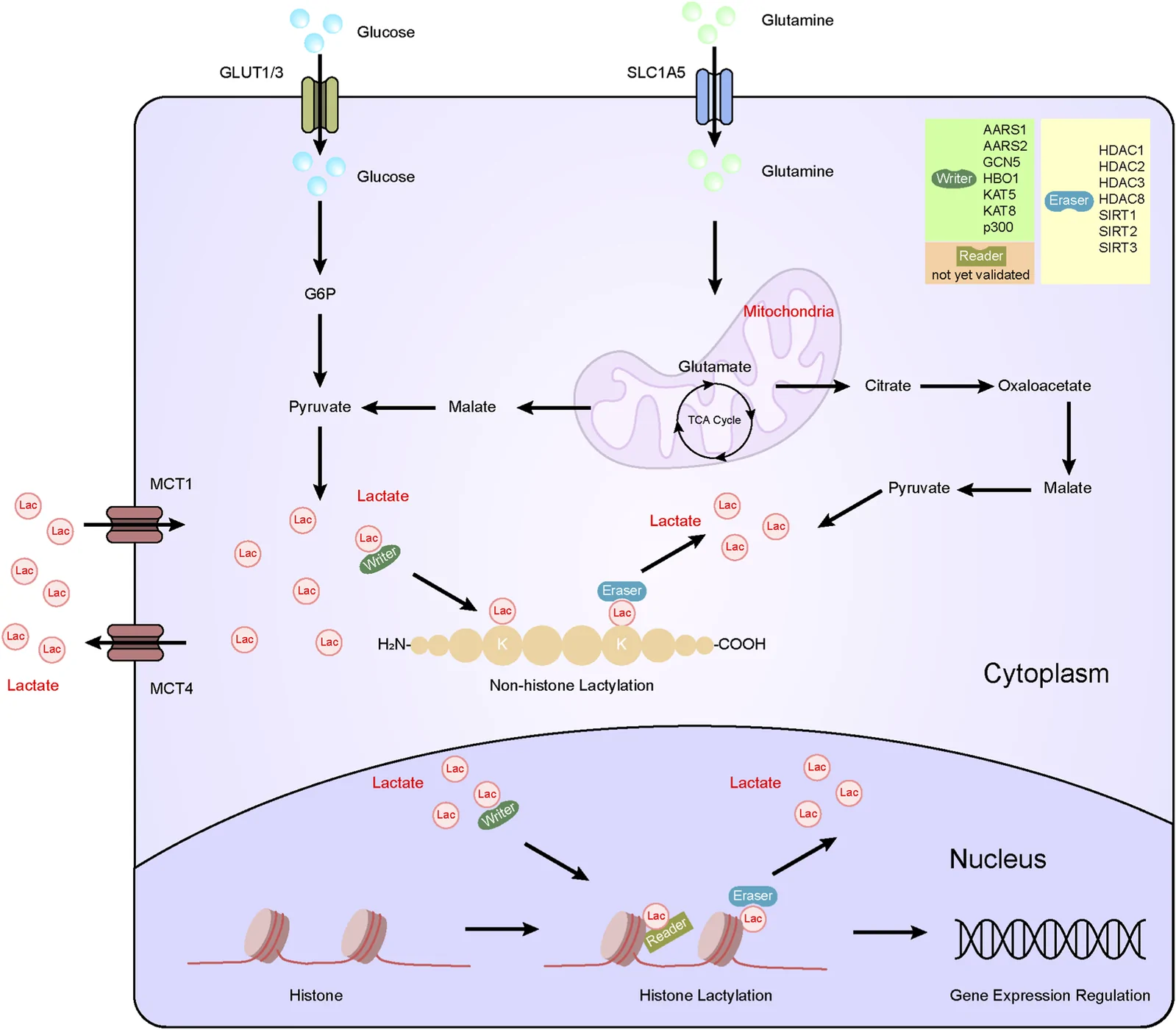
Integrated framework of histone and non-histone lactylation.

#### 2.2.1 Acyl-donor supply: from lactate to nuclear lactyl-CoA

How lactate is converted into activated acyl donors to participate in enzymatic Kla modification is a core biochemical question, and its generation pathway and compartmentalization regulation are still under investigation. However, glioma-related studies have confirmed that nuclear lactyl-CoA can be continuously synthesized to maintain histone lactylation under metabolic stress. For example, nuclear GTPSCS, as a lactyl-CoA synthetase, interacts with p300 to elevate H3K18 lactylation and drive pro-tumor transcriptional outputs linked to tumor growth and radioresistance (Liu R. et al., 2025).

Meanwhile, oncogenic signals interact with the lactylation regulatory machinery to strengthen the lactate-to-chromatin link. For instance, the EGFR-driven pathway regulates histone lactylation through enzymatic coupling of lactyl-CoA-related metabolism and chromatin-modifying activity, in which ACSS2-mediated nuclear metabolism cooperates with KAT2A to install H3K14la/H3K18la modifications, and then initiates immune-evasion transcriptional programs such as PD-L1 expression (Zhu R. et al., 2025). These findings build a mechanistic bridge between lactate availability in glioma and the stabilization of chromatin states.

#### 2.2.2 Writers: enzymatic installation of lactyl marks

Intracellular Kla levels correlate with the abundance of lactate and lactyl-CoA, and available evidence supports the existence of enzymatic “writers” that install lactyl groups at specific sites. Histone acetyltransferases (HATs), especially p300/CBP and KAT2A, are considered candidate lactyltransferases, suggesting that lactylation shares a regulatory logic similar to that of other lysine acylation modifications, in which the deposition and turnover of modifications are controlled by specific enzymes (Zhu R. et al., 2025).

However, the enzymology of lactylation remains controversial. Many studies suggest that when local acyl donors are highly abundant, a subset of intracellular Kla may also arise through metabolite-driven chemical reactions (Gaffney et al., 2020). In addition, *in vitro* experiments indicate that the lactyltransferase activity of p300/CBP is weaker than its canonical acetyltransferase activity, so it cannot be assumed that all Kla changes are enzyme-dominated. Nonetheless, glioma-related studies support a functional role for regulated writer activity, as perturbation of p300/CBP or KAT2A alters global and/or site-specific Kla levels and is accompanied by changes in malignant phenotypes and therapeutic responses. Current evidence therefore suggests that, in the lactate-rich microenvironment of glioma, enzymatic writing contributes to the formation of a functional Kla regulatory network, whereas the relative contribution of enzymatic versus non-enzymatic pathways likely depends on cellular compartment and substrate context.

#### 2.2.3 Erasers: delactylation and its implications for therapy response

Kla is recognized as a reversible modification, which means the existence of enzymatic “erasers” that remove lactyl groups, and this has direct translational significance in glioma research because delactylation can inhibit transcriptional programs associated with chemoresistance (Moreno-Yruela et al., 2022). A typical example is SIRT6-mediated H3K9 delactylation, which suppresses MGMT transcription and reverses temozolomide resistance. This builds a clear mechanistic association between dynamic Kla changes and the classical determinant of temozolomide response, and also makes delactylation a potential therapeutic target (Zou et al., 2025).

#### 2.2.4 Readers and downstream interpretation of lactylation signals

In principle, Kla marks may exert downstream effects through proteins that selectively recognize lactylated lysine residues and couple these modifications to transcriptional or signaling outputs. However, reader proteins remain the least established component of the Kla regulatory machinery, particularly in glioma. Importantly, no Kla reader has yet been directly validated in glioma through combined biochemical recognition and functional analyses.

Evidence for Kla readers currently comes primarily from non-glioma systems. Brg1 was reported to interact with H3K18la and participate in lactylation-dependent transcription during early induced pluripotent stem cell reprogramming. More recently, DPF2 was identified as an H3K14la effector through proteomic, biochemical, structural, and functional analyses in a non-glioma tumor context (Zhai et al., 2024). These studies establish the principle that histone Kla can be interpreted by dedicated effector proteins, but their relevance to glioma remains untested. By contrast, YEATS-domain and BET-family proteins are better regarded as established readers of other acyl-lysine modifications or as putative Kla-interacting factors, because direct evidence for selective Kla recognition remains insufficient (Zhao et al., 2016; Dey et al., 2003).

Therefore, the identity of physiologically relevant Kla readers in glioma remains an important unresolved question. Determining whether Brg1, DPF2, or other chromatin-associated proteins directly recognize Kla in glioma cells will require biochemical binding assays, structure-guided mutagenesis, chromatin occupancy analyses, and functional rescue experiments in glioma-specific models. Establishing such readers would fill an important mechanistic gap between Kla deposition and downstream transcriptional responses.

### 2.3 Methodological considerations for lactylation research

Rigorous detection and causal validation are prerequisites for analysing the lactate-Kla axis in glioma, and research needs to take into account the limitations of multiple technologies and concepts. First, antibody-based detection is prone to deviations: the epitope specificity of commercial antibodies varies, cross-reactivity with other lysine acylation modifications is a common problem, and detection signals are affected by pre-analytical variables such as tissue fixation and sample processing. Without orthogonal validation, the results can only be interpreted as semi-quantitative (Egelhofer et al., 2011; Rothbart et al., 2015). Second, mass spectrometry (MS) has higher detection specificity and can achieve modification site mapping, but this technology requires adherence to strict analytical criteria, sufficient detection depth and independent validation, because the stoichiometry of post-translational modifications such as Kla in biological samples is usually low (Hansen et al., 2019). Third, lactate abundance is not equivalent to functional Kla levels. Rigorous causal inference requires perturbing lactate production/transport, modulating candidate writers/erasers through experiments, and using site-specific mutagenesis and rescue experiments to verify the function of modified sites, so as to distinguish functional Kla events from merely correlated metabolic signatures.

## 3 Lactylation in glioma progression

### 3.1 Histone lysine lactylation couples lactate stress to transcriptional programs in glioma

Histone Kla is the earliest identified and most representative form of protein lactylation and represents an important epigenetic regulatory layer linking metabolic states to transcriptional control in glioma (Li H. et al., 2024). In the microenvironment of high glycolysis and high lactic acid in glioma, this modification (histone lactylation) can transform metabolic stress into a stable transcriptional program and promote the malignant progression of the tumor (Zhao et al., 2026). Similar to histone acetylation, histone Kla is highly enriched in the transcriptionally active chromatin region and can regulate tumor-related stable transcription output (Galle et al., 2022). Existing studies have confirmed that histone lactylation promotes the progression of glioma through site-specific transcription activation of cancer-promoting programs. Preclinical research and clinical correlation evidence show that it plays a key role in maintaining the glioma stem cell-like phenotype and reshaping the immunosuppressive microenvironment. NF-κB signaling enhances glycolysis and histone lactylation in GSCs and increases expression of the long non-coding RNA LINC01127. LINC01127 subsequently cis-regulates MAP4K4 and reinforces JNK/NF-κB signaling, thereby promoting GSC self-renewal and tumor progression (Li et al., 2023). Another glioma-specific mechanism involves histone H4K8 lactylation, which transcriptionally upregulates NUPR1 and promotes autophagosome-lysosome fusion and protective autophagic flux. This H4K8la-NUPR1 axis has been associated with enhanced glioblastoma proliferation, invasion, and tumor growth, suggesting a potential vulnerability to NUPR1 inhibition (Zhao et al., 2026).

The biological significance of Kla is also likely to vary across molecularly defined glioma subtypes. Most mechanistic studies to date have focused on adult IDH-wild-type GBM, which is typically characterized by pronounced glycolytic activity and a lactate-rich microenvironment. By contrast, IDH-mutant gliomas generally exhibit lower lactate levels than IDH-wild-type tumors and accumulate the oncometabolite 2-hydroxyglutarate, thereby establishing a distinct metabolic and epigenetic state (Wenger et al., 2020). Findings obtained in IDH-wild-type GBM therefore should not be directly extrapolated to IDH-mutant disease, for which direct functional studies of Kla remain scarce.

A distinct lactylation-associated program has also emerged in H3K27M-mutant diffuse midline glioma. In this setting, lactate-dependent NME1 K49 lactylation enhances nucleoside diphosphate kinase activity and nucleotide biosynthesis, thereby supporting tumor proliferation (Batsios et al., 2026). These observations suggest that Kla may involve subtype-specific substrates and biological outputs rather than representing a uniform metabolic response across gliomas. Systematic comparisons of Kla abundance, substrate repertoires, and functional dependencies across IDH-wild-type, IDH-mutant, and H3K27-altered gliomas are therefore needed. Importantly, histone Kla is also dynamically remodeled under treatment stress, and representative roles in TMZ adaptation are discussed in Section 4.

### 3.2 Non-histone Kla in protein stability, signaling, and metabolic adaptation

Although histone lactylation is an important level of transcription regulation, the lactylation in glioma is not restricted to chromatin. A growing body of evidence suggests that non-histone Kla acts as a more rapid and direct post-translational mechanism through which lactate-rich metabolic stress is translated into protein-state adaptation (Zhang et al., 2025; Gaffney et al., 2020). Compared with histone lactylation, non-histone lactylation can more directly affect enzyme activity, protein stability, subcellular localization and macromolecule interaction. In glioma, it participates in metabolic reprogramming, DNA damage repair, RNA processing, proteostasis and other processes. It is critical for rapid cellular stress responses in tumor cells exposed to nutrient and oxygen fluctuations or therapeutic stress.

Several representative substrates illustrate the functional diversity of non-histone Kla in glioma. X-ray repair cross-complementing protein 1 (XRCC1) K247 lactylation enhances DNA repair capacity and contributes to therapeutic resistance (Li G. et al., 2024), whereas Ku70 K317 lactylation links TAM-derived lactate to suppression of cGAS–STING signaling and immune escape (Li et al., 2026). In metabolic regulation, PTBP1 lactylation enhances its RNA-binding activity and stabilizes PFKFB4 mRNA, thereby reinforcing glycolytic flux and metabolic plasticity in GSCs (Zhou Z. et al., 2025). In H3K27M-mutant diffuse midline glioma, NME1 K49 lactylation enhances nucleoside diphosphate kinase activity and nucleotide biosynthesis, thereby supporting tumor proliferation and providing a potential metabolic imaging target (Batsios et al., 2026). In addition, histone H3K18 lactylation upregulates USP4 expression, after which USP4 deubiquitinates and stabilizes ANXA2, linking histone lactylation to ubiquitin-dependent proteostasis and radioresistance (Tu et al., 2025). Another example linking non-histone lactylation to metabolic plasticity is c-Myc. In glioblastoma cells, dexmedetomidine was reported to suppress migration, invasion, and glycolysis by reducing c-Myc lactylation and destabilizing c-Myc, further supporting the idea that non-histone Kla can directly modulate oncogenic metabolic regulators (Zhu and Zhang, 2024). Conceptually, non-histone Kla can therefore be understood as a transcription-independent interface between metabolism and protein function, complementing histone-based regulation by enabling more immediate shifts in signaling and protein activity.

### 3.3 Coordinated interplay between histone and non-histone Kla

Histone and non-histone lactylation are mutually coordinated but have different mechanisms of lactic acid-responsive regulation levels: the former mainly reshapes the transcriptional programs, while the latter directly regulates protein stability, signal pathway activity, metabolic enzyme function and macromolecule interaction. The two jointly transform the high lactic acid metabolic stress of glioma into a stable tumor malignant phenotype. This functional division of labour provides a plausible framework for glioma cells to combine slow transcription remodeling with rapid post-translational modification stress response.

Kla also operates within a broader network of post-translational modifications rather than functioning in isolation. A glioma-specific example is provided by the H3K18la–USP4–ANXA2 pathway. H3K18 lactylation enhances USP4 expression in GSCs, after which USP4 removes ubiquitin chains from ANXA2 and stabilizes the protein (Tu et al., 2025). In parallel, additional modifications of ANXA2, including acetylation and phosphorylation, can influence its interaction state and downstream signaling. This mechanism illustrates how a histone lactylation signal can be propagated through ubiquitination-, acetylation-, and phosphorylation-dependent protein regulation to reinforce GSC maintenance and radioresistance. Nevertheless, such mechanistically resolved examples remain uncommon in glioma. Crosstalk between Kla and other lysine acylations may arise through competition for the same lysine residues, shared use of acyltransferases or deacylases, or indirect transcriptional regulation. Future studies should distinguish direct site competition from pathway-level associations by combining site-specific mass spectrometry, combinatorial mutagenesis, and quantitative analysis of modification stoichiometry. To provide a comprehensive overview, the key histone and non-histone substrates of lysine lactylation in glioma, along with their specific modification sites and biological consequences, are summarized in Table 1.

**TABLE 1:** Major histone and non-histone lactylation events and their biological and therapeutic relevance in glioma.

Category	Lactylated target/site	Representative regulatory mechanism	Major biological function in glioma	Potential therapeutic relevance	Evidence level
Histone Kla	H3K18la	Generated through GTPSCS/p300-dependent lactyl-CoA utilization and activates downstream GDF15 and USP4–ANXA2 programs	Promotes GSC maintenance, tumor proliferation, stem-like properties, and radioresistance	Targeting nuclear lactyl-CoA synthesis, p300 activity and USP4/ANXA2 signaling may suppress malignant progression and improve radiosensitivity	*In vitro*; patient-derived GSC and *in vivo* validation in selected studies; clinical correlation; no prospective validation
Histone Kla	H3K9la	Activates LUC7L2 transcription, resulting in MLH1 intron retention and MMR impairment; H3K9 delactylation by SIRT6 suppresses MGMT transcription	Facilitates acquired TMZ resistance through impairment of MMR and activation of MGMT-associated resistance programs	Inhibition of lactate production with stiripentol or restoration of SIRT6-mediated delactylation may resensitize GBM to TMZ	*In vitro*; *in vivo* GBM models; retrospective clinical correlation; no prospective validation
Histone Kla	H4K8la	Transcriptionally activates NUPR1, promoting autophagosome–lysosome fusion and protective autophagic flux	Enhances GBM proliferation, invasion, tumor growth, and survival under metabolic stress	Targeting lactate production or inhibiting NUPR1 with ZZW-115 may disrupt the H4K8la–NUPR1–autophagy axis	*In vitro*; *in vivo*; human tissue correlation; no prospective validation
Non-histone Kla	XRCC1 K247la	Enhances XRCC1 binding to importin-α, nuclear translocation, and base excision repair	Accelerates repair of radiation-induced DNA damage and promotes intrinsic and acquired chemoradiotherapy resistance	D34-919, which disrupts the ALDH1A3–PKM2 axis upstream of lactate accumulation, may selectively radiosensitize ALDH1A3-high GBM	*In vitro*; *in vivo*; clinical correlation; no prospective validation
Non-histone Kla	Ku70 K317la	TAM-derived lactate is exported through MCT4 and imported into GSCs through MCT1, inducing Ku70 lactylation and suppressing cGAS–STING–type I interferon signaling	Promotes DNA damage tolerance, innate immune escape, an immunosuppressive TME, and TMZ resistance	Blocking MCT1/MCT4-mediated lactate transport or Ku70 K317la may enhance DNA damage signaling and improve responses to immune checkpoint blockade	Cell-based mechanistic studies; *in vivo* immunocompetent models; no prospective clinical validation
Non-histone Kla	PTBP1 K436la	Enhances PTBP1-dependent RNA regulation and reinforces PFKFB4-driven glycolysis	Sustains glycolytic plasticity, GSC self-renewal, and tumorigenic capacity	The PTBP1–PFKFB4 axis represents a potential metabolic vulnerability for disrupting GSC maintenance	*In vitro* and preclinical *in vivo* validation; no prospective clinical validation
Non-histone Kla	NME1 K49la	Activates NME1 nucleoside diphosphate kinase activity, increasing NTP biosynthesis	Promotes DNA replication, cell-cycle progression, and tumor proliferation in H3K27M-mutant diffuse midline glioma	Targeting the H3K27M–PGK1–lactate–NME1 axis may suppress nucleotide synthesis; deuterium metabolic imaging may enable longitudinal monitoring of tumor growth and treatment response	Preclinical H3K27M glioma models with *in vivo*/metabolic imaging validation; no prospective validation
Non-histone Kla	c-Myc lactylation *(site not specified)*	Increases c-Myc protein stability and sustains c-Myc-dependent glycolytic programs	Promotes glycolysis, migration, and invasion of GBM cells	Dexmedetomidine was reported to reduce c-Myc lactylation and stability, although the evidence is currently limited mainly to cell-based models	Primarily cell-based evidence; no *in vivo* or prospective clinical validation

## 4 Lactylation and therapeutic resistance in glioma

### 4.1 Therapy-induced lactate stress and Kla remodeling

The strength of evidence supporting Kla-mediated therapeutic resistance varies considerably among reported pathways. Some mechanisms have been supported by site-directed mutagenesis and animal models, whereas others remain based primarily on cell-based experiments or retrospective associations in patient specimens. Accordingly, mechanistic causality and clinical correlation should be distinguished from prospective therapeutic validation throughout this section.

Under treatment pressure, lactylation is not simply increased in a uniform manner; rather, radiotherapy and temozolomide reshape Kla in a context-, site-, and compartment-dependent fashion (Qiu et al., 2025; Yue et al., 2024). Therapy-associated lactate accumulation should not be interpreted as functionally equivalent to lactylation. Instead, treatment stress may influence Kla either directly by altering lactate/lactyl-CoA availability or indirectly by activating signaling pathways that reshape acyl-donor supply, enzyme localization, or chromatin accessibility. Radiotherapy can increase lactic acid production through hypoxia-related signals and stress-induced metabolic remodeling, which is especially obvious in the perinecrotic niches enriched for glioma stem-like cells. Treatment-associated Kla remodeling has also been observed in resistant cell models and retrospective clinical specimens. Altered H3K9la/H3K18la levels and changes in regulatory factors such as SIRT6 have been reported in individual studies (Zhao et al., 2016; Yip et al., 2009; Sun et al., 2023). However, these clinical observations remain largely correlative and require validation in larger, prospectively annotated cohorts.

### 4.2 Lactylation in temozolomide resistance

Emerging evidence indicates that lactylation adds an additional regulatory layer to established determinants of TMZ resistance, including MGMT activity and mismatch repair (MMR) integrity (Hegi et al., 2005; Yip et al., 2009; Cahill et al., 2007) (Figure 2). Lactylation does not work through a single linear mechanism, but links prolonged alkylation stress with chromatin remodeling, DNA damage adaptive changes and microenvironment-mediated survival programs, to promote the transformation of tumor cells from short-term drug tolerance to stable temozolomide-resistant states. Among the best-characterized examples is the H3K9la–LUC7L2–MLH1 axis, which links histone lactylation to impaired mismatch repair (Yue et al., 2024). Under prolonged TMZ exposure, increased H3K9la promotes LUC7L2 transcription, resulting in MLH1 intron retention and reduced MMR activity. This pathway is supported by mechanistic cell-based experiments, *in vivo* GBM models, and retrospective analyses of patient specimens. Pharmacological inhibition of lactate production with stiripentol enhanced TMZ sensitivity in preclinical models. However, prospective clinical validation of this therapeutic strategy is currently lacking.

**FIGURE 2:**
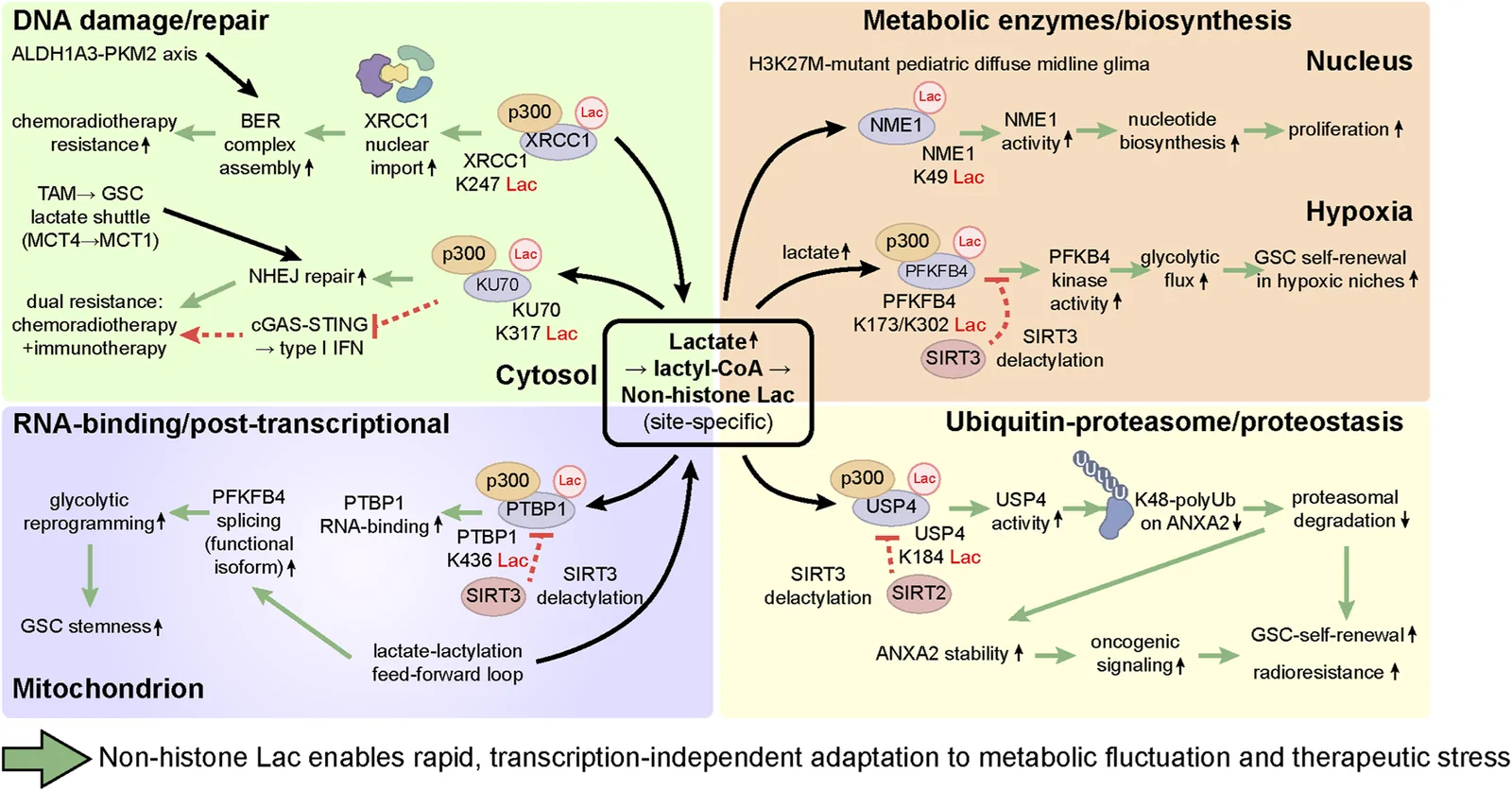
Lactylation-driven mechanisms of TMZ resistance.

A complementary line of evidence points to site-specific remodeling of histone lactylation during TMZ adaptation. In established TMZ-resistant GBM cell models, H3K18la is increased together with enhanced glycolytic activity. Pharmacological activation of PPARα reduces intracellular lactate availability and H3K18la, thereby improving TMZ sensitivity in preclinical experiments (Wang et al., 2025). These findings provide mechanistic and preclinical support, but clinical validation is currently lacking. The SIRT6–H3K9la–MGMT axis provides another example of lactylation-dependent TMZ adaptation. This mechanism is supported by TMZ-resistant GBM cell models and *in vivo* therapeutic experiments, together with clinical associations between reduced nuclear SIRT6 expression and poor outcome (Zou et al., 2025). Pharmacological activation of SIRT6 enhanced TMZ responses in preclinical models. However, no prospective clinical evidence currently demonstrates therapeutic benefit from SIRT6-directed modulation in GBM, and brain exposure, safety, and biomarker selection remain unresolved. In glioblastoma, lactic acid derived from macrophages is transported to GSCs through the MCT4-MCT1 axis, inducing its Ku70 K317 lactylation (Li et al., 2026). This regulatory program enhances the tolerance of tumor cells to treatment-induced DNA damage, inhibits inherent immune recognition, promotes temozolomide tolerance, and reinforces the immunosuppressive microenvironment, reducing the therapeutic effect. The TAM–MCT4/MCT1–Ku70 K317la pathway is supported by mechanistic GSC/TAM experiments and preclinical *in vivo* models. In these settings, disruption of lactate transport or Ku70 K317 lactylation enhanced innate immune signaling and improved responses to immune checkpoint blockade. Nevertheless, its predictive value and therapeutic efficacy in patients with GBM remain untested prospectively.

### 4.3 Lactylation in radioresistance and chemoradiotherapy adaptation

Lactylation also participates in radiation resistance and combined treatment tolerance of glioma through a variety of mechanisms and different pathways. Its mechanisms of action include: direct non-histone lactylation modification of DNA repair factors, maintaining stem cell-like programs for treatment tolerance, and maintaining the level of lactylation under treatment stress through compartmental metabolic-epigenetic coupling (Chen et al., 2024). Among the more extensively characterized mechanisms is XRCC1 K247 lactylation, which enhances base excision repair and contributes to resistance to radiation-induced DNA damage (Li G. et al., 2024). This mechanism is supported by studies in GSCs and *in vivo* GBM models. Retrospective clinical observations further associate elevated lactylation-related features with unfavorable responses following chemoradiotherapy. Pharmacological disruption of the upstream ALDH1A3–PKM2 axis with D34-919 enhanced radiosensitivity in preclinical models. However, prospective biomarker-guided validation of this strategy has not yet been performed.

The histone-dependent regulatory programs can also participate in radiation resistance by maintaining the stem cell-like state of treatment tolerance. Recent studies have shown that H3K18 lactylation-dependent USP4 upregulation can stabilize ANXA2 through deubiquitination, enhance the stem cell-like characteristics of tumor cells, and thus promote radiation resistance (Tu et al., 2025). Tumor cells can also produce adaptive changes through the regulation of intranuclear acyl donor supply: nuclear GTPSCS can function as a lactyl-CoA synthetase, which cooperates with p300 to enhance H3K18 lactylation and activate GDF15 and other cancer-promoting transcriptional programs (Liu R. et al., 2025). In addition, radiation-related lactylation changes also interact with tumor immune escape: the lactic acid shuttle of tumor-related macrophages-GSCs can enhance Ku70 lactylation, inhibit cGAS-STING-type I interferon axis, weaken intrinsic immune recognition, and reduce the therapeutic effect of radiotherapy combined with immune checkpoint inhibitors.

Overall, the current evidence linking Kla to therapeutic resistance in glioma remains predominantly preclinical. Several pathways have been validated using cell-based systems, patient-derived GSCs, and intracranial or immunocompetent animal models, while selected studies have incorporated retrospective patient specimens. However, prospective clinical validation is currently absent. Moreover, robust confirmation in glioma organoids and treatment-response PDX models remains limited for most Kla-dependent resistance mechanisms. Future studies should therefore prioritize clinically representative organoid and PDX systems, longitudinal sampling, and biomarker-stratified prospective cohorts before Kla-dependent resistance pathways can be considered clinically actionable.

## 5 Translational implications, biomarkers, and future directions

### 5.1 Targetable nodes and therapeutic strategies along the lactate-Kla axis

The lactate–Kla axis integrates metabolic reprogramming, chromatin regulation, DNA damage adaptation, and tumor–immune interactions in glioma, thereby providing several potential levels of therapeutic intervention (Figure 3). However, the lactate–Kla axis should not be considered a single drug target. Instead, its potentially actionable nodes can be broadly organized into three intervention levels. The first aims to restrict lactate production or intercellular transport. The second targets lactyl-donor supply or the enzymes responsible for Kla deposition and removal. The third focuses on downstream Kla-dependent effectors involved in tumor stemness, DNA repair, immune evasion, and oncogenic signaling. In practical application, the therapeutic value of these intervention strategies varies among patients, and they need to be used in combination with biomarkers and standard treatment, rather than applied alone. In the first level, the most direct strategy is to reduce lactic acid production or block lactic acid transport: in the preclinical model of glioblastoma, inhibiting LDH/LDHA can reduce lactic acid levels and weaken lactic acid-related transcriptional programs; blocking MCT-dependent lactic acid transport can disrupt the lactic acid shuttle from macrophages to GSCs, restore the activity of the cGAS-STING pathway, and enhance the anti-tumor immune effect (Li H. et al., 2024; Sun et al., 2023). However, targeting lactate production or transport carries important limitations. LDH and MCT family members have broad physiological functions in normal tissues, including the brain, muscle, and immune system, raising concerns regarding systemic metabolic toxicity and a potentially narrow therapeutic window. In addition, blockade of a single lactate-production or transport pathway may be partially compensated by metabolic plasticity and alternative carbon utilization. Tumor-selective delivery, pharmacodynamic monitoring, and adequate central nervous system exposure will therefore be essential for further therapeutic development.

**FIGURE 3:**
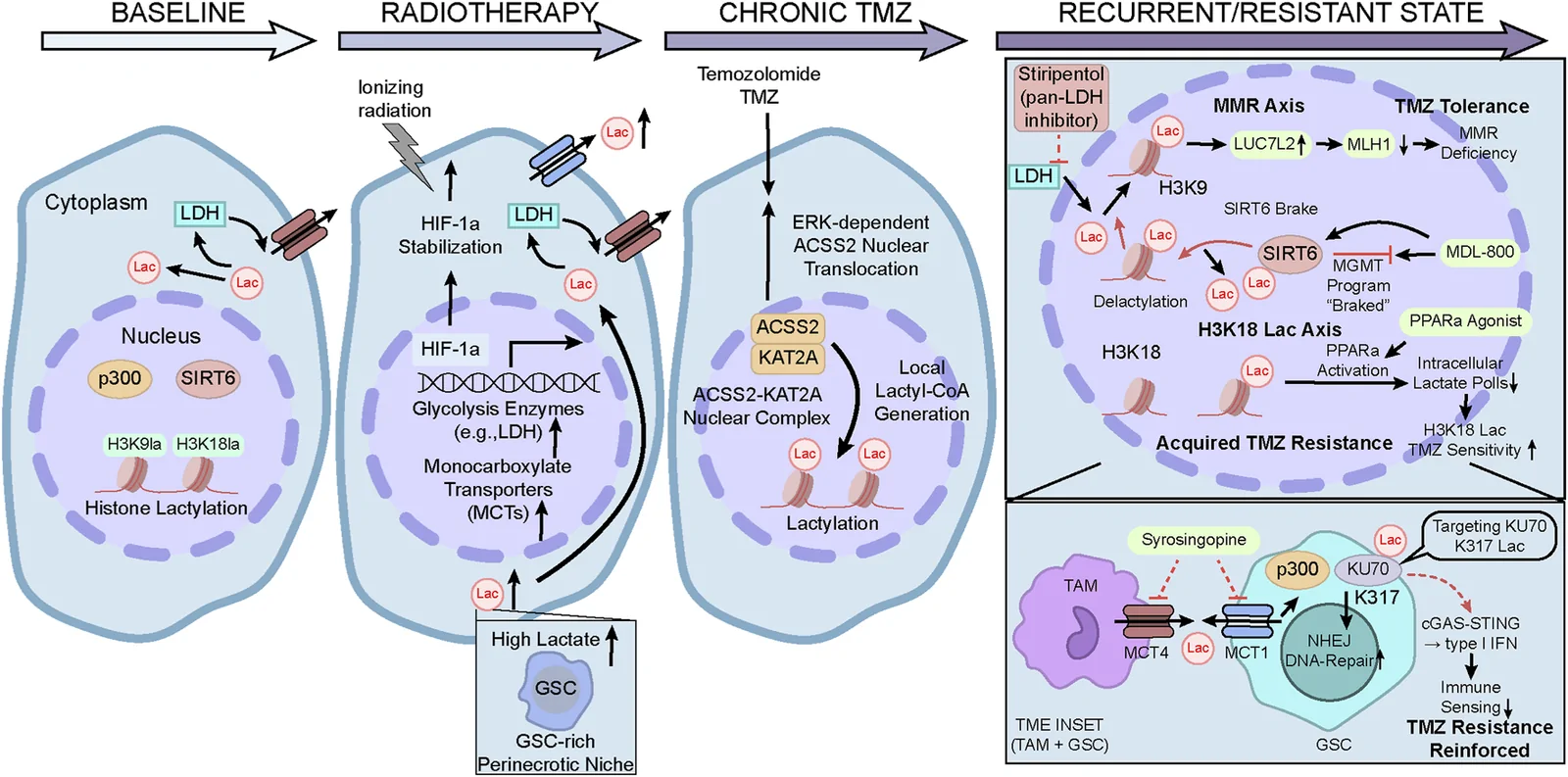
Therapy-induced evolution of lactate–Kla remodeling during glioma treatment adaptation.

The second level focuses on the biochemical regulation system that directly maintains histone lactylation. In glioblastoma, the availability of intranuclear lactyl coenzyme A is related to ACSS2 and GTPSCS. These two molecules can synergize with acetyl transferase-related systems to maintain histone lactylation and activate the cancer-promoting transcriptional programs. On the contrary, the delactylation pathway can be used as an endogenous drug resistance inhibition mechanism, such as SIRT6-mediated H3K9 delactylation, which can inhibit MGMT transcription and antagonize temozolomide resistance. Targeting the Kla regulatory machinery presents an additional challenge because most candidate enzymes are pleiotropic rather than Kla-specific. p300/CBP and KAT2A regulate multiple lysine acylation states and broad transcriptional programs, whereas ACSS2 and GTPSCS participate in metabolic processes beyond lactyl-CoA generation. Likewise, pharmacological modulation of sirtuins may affect numerous non-Kla substrates. Therefore, phenotypes produced by these agents cannot automatically be attributed to changes in Kla. Substrate-, compartment-, or site-selective modulators, together with pharmacodynamic evidence of specific Kla target engagement, will be required to improve therapeutic specificity.

The third level targets the downstream effect molecules maintained by the lactylation dependence programs, including the key nodes involved in oncogenic signaling, tumor stemness maintenance, RNA processing, DNA damage repair and immune escape. Downstream Kla-dependent effectors may offer greater mechanistic specificity, but this strategy is constrained by intratumoral heterogeneity, pathway redundancy, and the limited druggability of many currently identified substrates. Moreover, activity in peripheral or subcutaneous tumor models does not ensure adequate drug exposure in infiltrative glioma regions, because an intact or partially intact blood–brain barrier persists in substantial portions of GBM (Oberoi et al., 2016; Miller, 2010). These limitations must be considered when prioritizing downstream Kla effectors for translational development.

From the perspective of transformation research, the three intervention levels complement each other and can be integrated with the existing treatment plan: in temozolomide chemotherapy, the core of the lactylation targeting strategy is to block chromatin remodeling and DNA repair adaptive changes under prolonged alkylation stress; in radiotherapy and concurrent chemoradiotherapy, it can also target lactylation-dependent DNA repair enhancement and stem cell-like cells with treatment tolerance; in immunotherapy, the key is to break the lactic acid-driven immunosuppressive microenvironment, especially targeting the lactic acid transport and lactylation-dependent immune escape pathways. Importantly, no therapeutic strategy specifically targeting the Kla axis has yet undergone prospective clinical validation in glioma. Therefore, all Kla-directed therapeutic approaches discussed above should currently be regarded as preclinical or hypothesis-generating rather than clinically established treatment options.

### 5.2 Biomarkers and translational constraints

The lactate–Kla axis offers potential translational opportunities in glioma, but its clinical development faces several challenges specific to central nervous system tumors. Many candidate agents may have systemic toxicity or insufficient blood–brain barrier penetration. In addition, pronounced spatial and cellular heterogeneity limits the interpretability of bulk-tissue measurements. The lack of standardized and prospectively validated biomarkers further complicates patient selection and treatment monitoring (Sottoriva et al., 2013; Sarkaria et al., 2018; Schulz et al., 2023; Cagney et al., 2018). At present, the most mature research is the prognostic biomarker of lactylation-related genetic characteristics (Zhu H. et al., 2025). The retrospective analysis based on The Cancer Genome Atlas (TCGA), Chinese Glioma Genome Atlas (CGGA) and single-cell and spatial transcriptomics data indicates that this gene trait can be stably divided into risk groups with different biological and clinical characteristics of patients, but these results are still retrospective prognostic associative analysis and have not yet become a standardized clinical biomarker.

The research on predictive biomarkers for lactylation targeted treatment is not yet mature. XRCC1 K247la, Ku70 K317la and other site-specific non-histone lactylation markers can reflect the activation status of DNA repair adaptation and immune escape-related functional programs. They are potential mechanistic candidate markers, but most of the relevant data come from small sample retrospective cohorts or preclinical research. The expression state of lactylation regulatory factors, such as ACSS2, GTPSCS, p300/CBP, and SIRT6, can identify tumors that are highly dependent on specific lactylation regulatory nodes, but the association between the two is still in the preliminary research stage. Metabolic imaging technologies such as ^1^H-MRS and hyperpolarisation ^13^C-pyruvate MRS can capture the spatial and dynamic changes related to lactic acid and supplement the detection of tissue biomarkers, but the association between the imaging lactic acid signal and the specific lactylation programs is not clear (Booth et al., 2021; Zaccagna et al., 2022). In summary, the core challenge of current translational research is not to find more candidate markers, but to build a clinically applicable framework to correlate the lactylation biology, drug exposure and treatment responses in a repeatable way. Progress in this field requires standardized detection methods, prospectively designed biomarker research, and integrated molecular, spatial and metabolic test results in glioma cohorts with clinical annotations.

Taken together, the translational potential of the lactate–Kla axis extends beyond the inhibition of a single molecular target (Figure 4). Its clinical application will likely require coordinated intervention at the levels of lactate production and transport, the Kla regulatory machinery, and downstream lactylation-dependent effectors. These strategies may be integrated with standard chemoradiotherapy or immunotherapy and guided by molecular biomarkers, while the development of brain-penetrant agents remains a major prerequisite.

**FIGURE 4:**
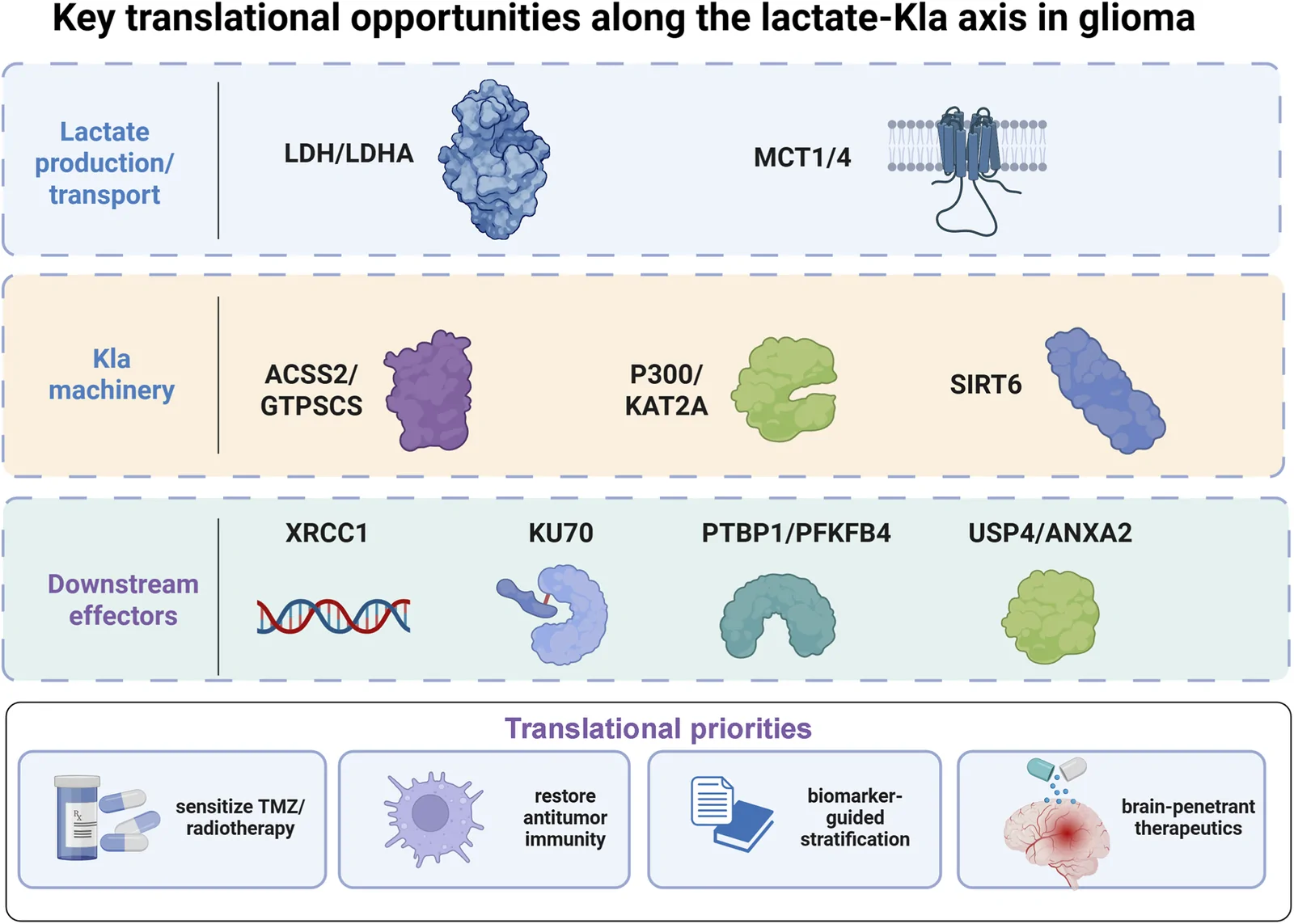
Key translational opportunities along the lactate–Kla axis in glioma.

All strategies shown remain preclinical and require further validation of safety, brain penetration, pharmacodynamic target engagement, and biomarker-guided patient selection before clinical translation.

### 5.3 Future directions

Although the research on glioma lactylation is progressing rapidly, this field is still in its infancy, and many problems limit the interpretation of the mechanism and clinical transformation. Future research should move beyond the descriptive cataloguing of lactylation-related events, focus on establishing a rigorous causal regulation framework, and clarify how the stress of lactic acid sources can be transformed into stable tumor malignant programs. At present, the main research gap is that the glioma-specific lactylation identification protein and downstream effect molecules have not been clarified. Current mechanistic evidence is heavily weighted toward adult IDH-wild-type GBM, whereas the lactate–Kla landscape in IDH-mutant glioma and pediatric H3K27-altered diffuse midline glioma remains incompletely characterized. Future studies should determine whether differences in metabolic state, oncometabolite accumulation, developmental context, and cell lineage generate subtype-specific Kla substrates and functional dependencies. In parallel, Kla should be investigated as part of a broader PTM network rather than as an isolated modification. Defining whether lactylation directly competes or cooperates with acetylation, ubiquitination, phosphorylation, and other modifications at specific substrates will be essential for understanding the context-dependent consequences of Kla in glioma.

At the level of translational research, lactylation targeted therapy still faces many limitations: lack of standardized and prospectively verified biomarkers; lack of small molecule drugs that can pass through the blood-brain barrier; the role of lactylation in treatment-driven tumor cloning evolution and continuous treatment tolerance state is still unclear. In general, the future development of glioma lactylation research depends on the accuracy of mechanism research, the biological research of tumor subtype specificity, the standardization of biomarkers, and the research and development of therapeutic drugs that can pass through the blood-brain barrier. The current core challenge is no longer whether lactylation is related to glioma, but to clarify the timing and scenario of the lactic acid–lactylation signaling axis, and which patients are therapeutically actionable.
